# An Improved *Theileria parva* Sporozoite Seroneutralization Assay for the Identification of East Coast Fever Immune Correlates

**DOI:** 10.3390/antib13040100

**Published:** 2024-12-05

**Authors:** Hannah Chege, Samuel Githigia, James Gathumbi, Naomi Chege, Rose Ojuok, Josiah Odaba, Stephen Mwalimu, Harriet Oboge, Lucilla Steinaa, Vishvanath Nene, Anna Lacasta

**Affiliations:** 1 Animal and Human Health Program, International Livestock Research Institute, Nairobi P.O. Box 30709, Kenya; h.chege@cgiar.org (H.C.); naomichege22@gmail.com (N.C.); r.ojuok@cgiar.org (R.O.); j.odaba@cgiar.org (J.O.); smwalimu@ufl.edu (S.M.); harriet.oboge@wsu.edu (H.O.); l.steinaa@cgiar.org (L.S.); v.nene@cgiar.org (V.N.); 2 Department of Veterinary Pathology, Microbiology and Parasitology, Faculty of Veterinary Medicine, University of Nairobi, Nairobi P.O. Box 29053-00625, Kenya; sgithigia@uonbi.ac.ke (S.G.); jkgathumbi@uonbi.ac.ke (J.G.)

**Keywords:** East Coast fever, *Theileria parva*, sporozoites, seroneutralization assay, antibodies, antigen

## Abstract

Background: Immune correlates of protection are ideal tools to predict treatment or vaccine efficacy. However, the accuracy of the immune correlate and the capability to robustly predict the outcome of a vaccine candidate are determined by the performance of the in vitro immunoassay used. Several *Theileria parva* sporozoite seroneutralization assays have previously been used to assess antibody functional activities; however, a common limitation has been the need for fresh material, target cells and sporozoites, and operator-to-operator bias. An improved assay represents a positive step toward overcoming challenges associated with variability and it might provide a more reliable means of establishing an immune correlate with protection after sub-unit vaccine administration. Methods: Herein, we describe key improvements, among them, (1) the use of frozen parasites and target cells to avoid batch-to-batch variations and (2) the development of a new assay read-out based on the detection of infected cells through flow cytometry, instead of the use of Giemsa staining and microscopic evaluation, in order to improve the reproducibility of the results. Results: The improved seroneutralization assay is not only able to detect the individual neutralizing capacity of antibodies; it also detects the additive effect of antibody combinations. Conclusions: This effect is described for the first time in *Theileria parva* and is of great interest for new antigen discovery and/or the epitope discovery of already known antigens like p67, opening a new avenue for the identification of ECF immune correlates of protection and the in vitro down-selection of new *Theileria parva* vaccine candidates, thereby contributing to reducing the use of animals in challenge experiments.

## 1. Introduction

East Coast fever (ECF), endemic to Central, Eastern, and Southern Africa, is a tick-borne disease in cattle caused by the parasite *Theileria parva*. It has been estimated to kill over one million cattle annually, although currently, the numbers could be higher, lowering the income and animal source food of livestock-dependent families [1,2].

Current control methods for the disease are (1) the use of acaricides with their inherent impact on the environment and the development of resistant ticks and (2) the Infection and Treatment Method (ITM) of vaccination, where a lethal dose of parasite is administered concomitantly with long-acting oxytetracycline. The latter poses several limitations, such as the high cost and difficulty of vaccine production, the generation of carrier status in vaccinated animals, the protection achieved being strain-specific, and the delivery not being simple, necessitating an ultracold supply chain and trained personnel [3]. To overcome the limitations of ITM vaccination, many efforts have been made to develop subunit vaccines using specific antigens targeting mainly two stages of the parasite life cycle, the sporozoite and the schizont. During the blood meal, ticks transmit the *Theileria parva* sporozoites into cattle. Sporozoites bind and enter bovine lymphocytes via a passive “zippering” process to the host cell membrane and differentiate into schizonts. The schizont-infected cells acquire a metastatic phenotype and are the primary cause of pathology. Even protection against ECF is mainly mediated by CD8^+^ T-cell lymphocytes with cytotoxic activity. ECF is a dose-dependent disease (meaning that the severity of the disease depends on the amount of infectious parasite transmitted), and the capacity to block sporozoite lymphocyte infection would provide a comparative advantage to the cattle to survive the disease (reviewed in [4]).

Cattle, rabbits, and mice re-infected several times with *T. parva* sporozoites developed neutralizing antibodies against the same sporozoite antigenic determinant. This antigen was identified as a ~70 kDa protein, and named p67 [5,6]. Although several other antigens have been used in the development of a subunit vaccine to block sporozoite infection, the surface antigen p67 remains the most promising one, providing 50% protection to vaccinated animals (reviewed in [4]). However, the role of p67-specific antibodies in protection after subunit vaccination is not clear. The diversity of in vitro assays used for both antibody titration and neutralization assays, together with the use of fresh material from different sources for every assay, has not helped to draw strong conclusions on the role of antibodies in protection against ECF [5,7,8,9]. Lacking a consistent immune correlate hampers the possibility of avoiding in vivo challenge experiments to screen antigens and the faster advance of vaccine development, and without a reliable in vitro assay, such a correlate cannot be found.

The advantage of having a strong in vitro assay correlating with protection is undeniable in the field of malaria, another parasitic disease, where the use of the Growth Inhibition Assay (GIA) has catapulted the screening of antigens and antigen delivery systems against the blood stage of the *Plasmodium* parasite. The GIA has been widely used to evaluate the vaccine-induced functional antibody activity of candidate antigens [10,11] and, as opposed to the field of *Theileria parva*, the GIA is a very well established protocol used by different reference labs across the globe and offered to the scientific community. However, the GIA also has many challenging variables; one of the most important is RBC donor variability in establishing synchronized infections. This is a common limitation in in vitro parasite neutralization assays, the lack of a cell line or large parasite batches to use consistently, thus the need to use primary cells from donors and fresh infectious material [12]. This fact highlights the complexity of developing parasite neutralization assays, in contrast to more simple approaches for viral in vitro assays, where cell lines are available, there is no large batch-to-batch variation in the infectious material, and the simplicity of the life cycle and infection process makes possible the development of surrogate neutralization assays [13]. Although facing multiple challenges in setting up parasite neutralization assays, like in the malaria field using the GIA, the development of more consistent antibody functional assays for the development of vaccines targeting the sporozoite stage of *Theileria parva* is a key component to fast-track antigen screening and the down-selection of antigen delivery systems.

Herein, we describe the improvement of the *Theileria parva* sporozoite neutralization assay. Key improvements have been achieved, among them (1) the use of frozen parasites and target cells to avoid batch-to-batch variations and (2) the development of a new assay read-out based on the detection of infected cells through flow cytometry, instead of the use of Giemsa staining and microscopic evaluation used previously, in order to improve the reproducibility of results. The improved seroneutralization assay is not only able to detect the individual neutralizing capacity of antibodies, but it can also detect the additive effect of different combinations of antibodies, an effect not described before in *Theileria parva* and of great interest for vaccine antigen discovery.

## 2. Materials and Methods

### 2.1. PBMC Population Profile and Schizont-Infected Cell Detection

PBMCs were isolated as previously described [14] and frozen at a concentration of 2 × 10^7^ cells/vial in freezing medium (fetal calf serum (FBS, Gibco, Paisley, Scotland) complemented with 10% DMSO (Merk, Darmstadt, Germany)). PBMC subpopulations from frozen stocks were analyzed by means of cell surface staining and flow cytometry. Monoclonal antibodies (mAbs) were used to identify CD4^+^ (ILA11, [15]), CD8^+^ (IL-A51, [16]), and CD3^+^ (MMIA, [17] T-lymphocytes, as unpurified ascites diluted at 1/500, as previously described [18]. Goat anti-mouse IgG:FITC (clone: poly4053, Biolegend, San Diego, CA, USA), diluted at 0.5 μg/mL in FACS buffer (sterile PBS complemented with 2% FBS (Gibco) and 0.2% sodium azide (NaN3, Merk)), was used as a secondary antibody. The PBMC population profile was assessed in five batches of cells over 18 months.

Detection of *T. parva*-infected PBMCs was performed by means of intracytoplasmatic staining, as previously described [19]. Briefly, the cells were fixed with 1% paraformaldehyde (PFA, Merk) for 10 min at room temperature. Fixed cells were permeabilized for 30 min at room temperature in permeabilization buffer (PBS complemented with 10% FBS and 0.2% saponin (Merck); from hereon PBS-SAP). Infected cells were detected using a purified anti-polymorphic immunodominant molecule (PIM) monoclonal antibody (ILRI clone ILS40.2) [20] at 0.2 μg/mL in PBS-SAP, and the same goat anti-mouse IgG:FITC was used as a secondary antibody diluted in PBS-SAP. Established *Theileria parva* Muguga (TpM)-infected cell lines were used as a positive control for FACS staining. Sample data were acquired via flow cytometry using FACS Canto II (BD Biosciences, Erembodegem, Belgium). A gate on live cells was applied on forward scatter (FSC) and side scatter (SSC), and a minimum of 10,000 events were analyzed. Generated data were analyzed using FlowJo software (version 10.8.1, BD Life Sciences, Franklin Lakes, NJ, USA) and RStudio version 2024.9.0.375 (Boston, MA, USA).

### 2.2. Sporozoites Production and Long-Term Storage

Sporozoites from *Theileria parva* sporozoites (3087 stabilate) were generated by the ILRI Tick Unit, essentially as previously described [21]. The infection rate of collected ticks was evaluated via microscopy after staining the dissected tick salivary glands with Schiff reagent (BDH Chemicals, Radnor, PA, USA). A summary of the infected rate and the estimated sporozoites is provided in Table 1. Sporozoites were isolated from the tick salivary glands by grinding them with a pestle and mortar in sterile conditions and gently ground in a circular motion on ice until forming a fully homogenized solution. The solution was centrifuged at 200× *g* for 15 min at 4 °C to remove debris. The volume of the resultant supernatant containing the live *Theileria parva* sporozoites was measured and mixed with an equal volume of RPMI complemented with 15% FBS and 10% DMSO. The sporozoite batch was aliquoted into cryotubes containing equal volumes and stored at −80 °C overnight and then transferred into liquid nitrogen for long-term storage.

### 2.3. Antibody Purification and Quantification

Previously generated ascites were obtained from the ILRI biorepository. Monoclonal antibodies previously described as neutralizing, AR22.7 and 1A7 [9], were purified from ascites using the ImmunoPure^®^ Melon^TM^ Gel IgG Spin Purification Kit (Thermo Fisher Scientific, Waltham, MA, USA), following the manufacturer’s instructions. The bovine anti-p67C polyclonal sera (Bov-p67C) was purified for p67C-specific antibodies as previously described [22]. The antibodies were quantified using Qubit (Invitrogen, Waltham, MA, USA).

### 2.4. Sporozoite Infectivity Assay and Seroneutralization Assay

Frozen PBMCs from BL093 Holstein Friesian cattle (*Bos taurus*) were thawed, washed and cultured overnight in complete RPMI (cRPMI, RPMI complemented with 10% FBS (Gibco), 2 mM L-glutamine, 1 μg/mL gentamycin (Carl-Roth, Karlsruhe, Germany), 100 UI/mL penicillin (Merk), 100 μg/mL streptomycin (Merk) and 5 × 10^−5^ M 2-mercaptoethanol (BDH)) at 37 °C and 5% CO_2_. After incubation, the cells were washed once with cRPMI, counted using a hemocytometer, and diluted at 10^6^ PBMC/mL. Monoclonal antibodies (mAb) AR22.7 and 1A7 and polyclonal Bov-p67C were diluted at 20 and 2 μg/mL in cRPMI. The antibodies were used individually or in combination to evaluate additive/synergistic or antagonistic effects.

Fifty microliters of diluted antibodies was dispensed into a 96-well round-bottomed plate (Corning, 3799, New York, NY, USA). Frozen *Theileria parva* Muguga 3087 sporozoites were thawed using a water bath at 37 °C and immediately diluted in cold cRPMI at 1/20 for the seroneutralization assay and 1/20, 1/50, and 1/100 for the sporozoite infectivity kinetic assay. Fifty microliters of sporozoites were layered on top of the antibodies (final volume 100 μL/well and final concentration of antibodies 10 and 1 μg/mL). The mixture was then gently mixed every 10 min for a total of 30 min at room temperature. PBMCs at 10^5^ cells/well (100 μL/well) were then dispensed on top of the sporozoite–antibody and mixed gently. The cells were incubated at 37 °C and 5% CO_2_ for 12 days for the neutralization assay and from 8 to 12 days for sporozoite infectivity. Media were not changed during the incubation period. An infection control without the presence of antibodies (positive control for infection) and non-infected PBMCs (negative control for infection) were also included. All conditions were assessed in triplicate. The results are expressed as the percentage of the infection reduced by specific antibodies and concentration compared to the infection control. Generated data were analyzed using FlowJo software (version 10.8.1, BD Life Sciences) and GraphPad Prism version 10.4.0 (San Diego, CA, USA).

## 3. Results

### 3.1. The Peripheral Blood Mononuclear Cell Profile for BL093 Does Not Significantly Change over Time

The sporozoite seroneutralization assay has three key elements: target cells (bovine lymphocytes), *Theileria parva* sporozoites, and the neutralizing antibodies. Bovine PBMCs were selected as target cells since they are easy to isolate, survive freeze–thawing cycles, are easy to culture in vitro, and can be harvested in large quantities from a single cattle donor. The population profile of different PBMC batches was evaluated over time on five batches of PBMCs collected over a period of 18 months. The population profile included the detection of CD3^+^, CD4^+^, and CD8^+^ T-lymphocytes. All cell types were within the normal range for cattle [23]. Most importantly, for the seroneutralization assay, the values for all cell populations were consistent over time (Table 2).

### 3.2. Selection of the Best Sporozoite Dilution and Time of Infection

**Figure 1:**
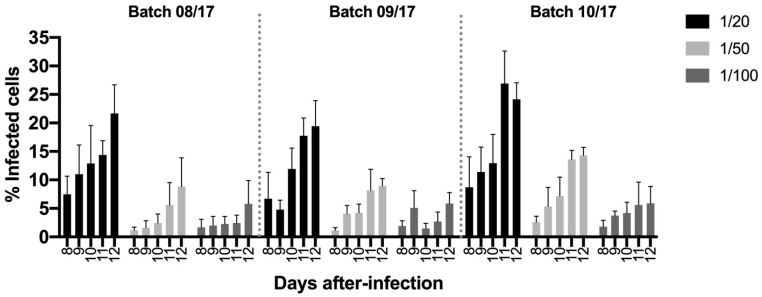
The percentage of infected cells was monitored using intracellular staining from day 8 to 12. The mean (column) and standard deviation (bars) are represented.

The second key element of the seroneutralizaton assay is the infectious agent, the *T. parva* sporozoites. With the aim of determining the best sporozoite dilution and time of infection, three batches of sporozoites (08/17, 09/17, and 10/17) were used for infectivity assays at different dilutions (1/20, 1/50, and 1/100) and incubation times (from 8 to 12 days post-infection). Pooled data from four assays are presented in Figure 1, where it can be observed that there was an increase in the number of infected cells from day 8 to day 12 in all of the sporozoite batches. A very low percentage and variable number of infected cells were detected until day 8 in the three sporozoite batches. The infection rate using 1/50 to 1/100 dilutions was not optimal, even after 12 days of incubation, ranging from 0.08 to 15.20% in the three batches. The dilution one in twenty presented a better scenario, with infection rates ranging from 16.2 to 28.9% for 08/17; from 11.2 to 24.0% for 09/17, and from 21.4 to 27.1% for 10/17 on day 12 (Figure 1 and Table S1 in Supplementary Materials). The time point with more consistent results was day 12 and was selected for the seroneutralization assay.

### 3.3. Assessment of the Neutralizing Capacity of Anti-p67 Antibodies Individually and in Combination

**Figure 2:**
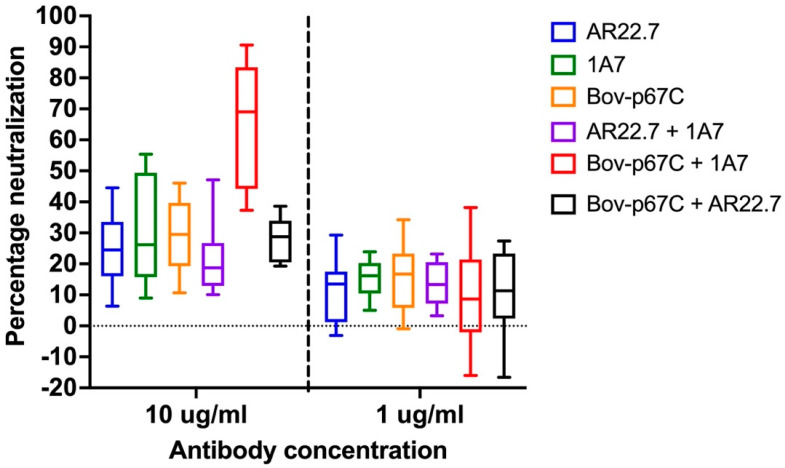
The neutralizing capacity of antibodies was assessed individually and in combination. The median and lower and upper quartile are represented by the box and the minimum and maximum values are represented by a vertical line.

The final key factor in the seroneutralization assay is the antibodies. Two mAb, 1A7 and AR22.7, and a polyclonal Bov-p67C, all against the major surface sporozoite antigen p67 and with neutralizing capacity [9], were selected for the assay. Five neutralization assays were performed using the antibodies individually and in combination to assess their capacity to neutralize sporozoite infection (Tables S3 and S4 in the Supplementary Materials). All mAbs or polyclonal antibodies presented some degree of neutralization at both concentrations, 10 μg/mL and 1 μg/mL, with medians ranging from 18.73 to 69.05% at 10 μg/mL and from 8.66 to 16.7% at 1 μg/mL (Figure 2 and Table S2 in the Supplementary Materials). As expected, the neutralizing capacity at 10 μg/mL was significantly higher than at 1 μg/mL in all cases; only the combination of AR22.7 and 1A7 showed no significant differences when comparing 10 to 1 μg/mL.

Interestingly, with this assay, we can also detect an additive neutralizing effect, as seen for the combination of the polyclonal Bov-p67C with the mAb 1A7, where the neutralizing capacity increased slightly more than 2 times compared with the median of the neutralizing capacities of the antibodies individually (*p*-values < 0.001 in both cases using the Mann–Whitney non-parametric test). The neutralizing capacity of the combination of Bov-p67C and mAb 1A7 is significantly higher than any other individual or combination of antibodies at 10 μg/mL (*p*-values < 0.0005 in all cases, Table S3 Supplementary Materials). The individual neutralizing capacity and the additive effect (69.05 % neutralizing capacity) make these two antibodies, Bov-p67C and 1A7, the best controls for the seroneutralization assay. No antagonistic effect was detected with the selected antibodies, but it remains a possibility that this could happen with other antibody combinations.

## 4. Discussion

Immune correlates of protection are ideal tools to predict treatment or vaccine efficacy. However, the accuracy of the immune correlate and the capability to robustly predict the outcome of a vaccine candidate are determined by the performance of the in vitro immunoassay used. Antibody functional assays, such as neutralization assays, are broadly used for antigen screening and to predict vaccine efficacy. One of many examples is SARS-CoV-2, where neutralization assays have been used to predict the severity of the disease in COVID-19 patients, the efficacy of monoclonal or synthetic antibodies as treatment tools, and vaccine efficacy against existing and new variants of the virus [24].

Unfortunately, and despite the fact that surviving cattle develop neutralizing antibodies against *T. parva* [5], a consistent correlate is missing for subunit vaccines priming antibodies to the sporozoite stage of the parasite. Although several neutralization protocols have been developed, from using live cattle reactivity to disease after the neutralization step to in vitro assays with tedious read-outs based on Giemsa staining and microscopy [8], a limitation in all of these assays is the need for fresh material, leading to batch-to-batch variations, and the operator bias intrinsic of the chosen read-outs, thus limiting assay repeatability and reproducibility. Solutions to these challenges were addressed in a more recent publication [7], where frozen target cells and sporozoites were used, but the assay needed many replicates to provide robust data, leading to a new limitation.

In the present work, we addressed all of these challenges and developed a robust *T. parva* sporozoite antibody neutralization assay. The use of frozen material, target cells (PBMCs), and infectious material (sporozoites) avoids batch-to-batch variations and allows for the analysis of up to 1000 samples in triplicate with a single lot of PBMCs and sporozoites, without introducing any new variables. Moreover, the use of frozen material avoids the constant use of live animals as PBMC donors and as “factories” to generate new batches of sporozoites [21], important factors to comply with the three Rs of animal use and welfare.

Preventing operator bias and reducing the number of replicas needed to obtain robust results was one of the main objectives of this work, and the detection of infected cells using flow cytometry addressed both challenges, totally avoiding operator-to-operator subjectivity and reducing the number of replicas from 10 to 3 per sample.

The neutralizing capacity of combinations of antibodies has not been assessed before, and it is of utmost importance for epitope and/or antigen screening. The improved assay has the capacity to assess the additive/synergistic activity of antibodies targeting different epitopes of the same antigen (1A7 + Bov-p67C), and as a result, it should be possible to assess antagonistic or competitive effects. In line with this, the assay can be used in a quantitative manner, allowing one to calculate the antibody concentration, providing a specific neutralizing value (e.g., 50% neutralization), as seen by the differences in the neutralizing capacity of the tested antibodies at different concentrations.

The analysis of many samples with a reliable assay will help to find an antibody immune correlate for ECF, as this happened when a correlation between protection and the growth inhibition capacity of antibodies against different *Plasmodium* antigens was identified. The Growth Inhibition Assay (GIA) helped to assess preclinical and early-clinical blood-stage vaccine candidates, with as many as 4 different antigens under evaluation, namely apical membrane antigen 1 (AMA-1), merozoite surface protein 1 (MSP1), erythrocyte binding antigen 175 (EBA-175), and the reticulocyte-binding protein homolog 5 (RH5) [11]. Malaria has greatly benefited from a robust and reliable in vitro assay for both, to identify an immune correlate and to screen antigens and delivery systems. Interestingly, we were able to detect the neutralizing capacity of anti-p67 monoclonal and polyclonal antibodies in the range of micrograms, while milligrams have had to be used in the malaria field using GIA to detect growth inhibition. The avidity of the antibodies to the parasite or the role of the antibodies during active infection might be plausible explanations. *Plasmodium falciparum* is not the only example of Apicomplexan parasites where antibody assays assessing the inhibition of infection have been used to identify putative protective antigens. Eimeria spp. are another example where antigen-specific antibodies were passively transferred into chicks to assess their capacity to block infection (reviewed in [25]). Another example is *Toxoplasma gondii*, where the use of infection inhibition assays allowed for the identification of the major surface antigen (SAG-1, P30) as a vaccine candidate [26,27,28].

In any case, ECF subunit vaccine development will benefit from having an improved seroneutralization assay able to analyze as many as 1000 samples per lot of material, narrowing down to the relevant epitopes in already known antigens and identifying new protective antigens in the more than 2007 proteins expressed in the sporozoite stage of the parasite [29].

## 5. Conclusions

We successfully developed a more robust *Theileria parva* seroneutralization assay that offers accuracy and efficiency in obtaining results while minimizing animal usage, overcoming many of the limitations that previous assays have faced. The assay is able to evaluate the neutralizing capacity of individual and combinations of antibodies and for the first time detect the additive/synergistic effects of antibody combinations. The new seroneutralization assay will open a new avenue to assess ECF immune correlates of protection with an antibody functional assay, leading to a more rational vaccine design.

## Figures and Tables

**Table 1:** Summarised information of the sporozoite batches generated.

Batch Name	Infected Acini/DSG *	Num. DSG *	Approx. Sporoz./Batch **	Num. Sporoz./Vial
08/17	50	500	10^9^	1.25 × 10^7^
09/17	50	550	1.1 × 10^9^	1.37 × 10^7^
10/17	50	503	10^9^	1.25 × 10^7^

* DSG: dissected tick salivary gland. ** Assuming an average of 40,000 sporozoites per infected acini.

**Table 2:** The percentage of cell subtypes was analyzed using flow cytometry using FACS Canto-II.

Batch Number	T-Lymphocytes (CD3^+^)	CD4^+^ T-Cells	CD8^+^ T-Cells
Batch 1 (16 March 2021)	36.7	15.8	23.5
Batch 2 (8 August 2021)	43.2	20.7	19.2
Batch 3 (3 February 2022)	36.2	13.4	19.1
Batch 4 (11 August 2022)	35.5	22.9	24.5
Batch 5 (4 October 2022)	48.8	11.6	21.8
Mean (95% CI *)	40.0 (34.8–45.1)	16.4 (12.8–20.1)	21.4 (19.5–23.3)

CI * = confidence interval 95%.

## Data Availability

The original contributions presented in this study are included in the article/Supplementary Materials. Further inquiries can be directed to the corresponding author.

## Supplementary Materials

The following supporting information can be downloaded at: https://www.mdpi.com/article/10.3390/antib13040100/s1. Table S1: Average percentage of T. parva-infected cells (and standard deviation) in the three sporozoite batches: 08/17, 09/17, and 10/17. Table S2: Neutralizing capacity of antibodies assessed individually and in combination at two different dilutions, 10 and 1 μg/mL. The statistical significance of the differences between 10 and 1 μg/mL was calculated using the Mann–Whitney non-parametric test (significant differences are in bold). Table S3: Statistical analysis of differences in the neutralizing capacity of individual and combinations of antibodies at 10 μg/mL, calculated using the Mann–Whitney non-parametric test.

## References

[1] McLeod A., Kristjanson R. (1999). Impact of Ticks and Associated Diseases on Cattlein Asia, Australia and Africa.

[2] Mukhebi A.W., Perry B.D., Kruska R. (1992). Estimated Economics of Theileriosis Control in Africa. Prev. Vet. Med..

[3] Perry B.D. (2016). The Control of East Coast Fever of Cattle by Live Parasite Vaccination: A Science-to-Impact Narrative. One Health.

[4] Nene V., Kiara H., Lacasta A., Pelle R., Svitek N., Steinaa L. (2016). The Biology of Theileria Parva and Control of East Coast Fever—Current Status and Future Trends. Ticks Tick Borne Dis..

[5] Musoke A.J., Nantulya V.M., Buscher G., Masake R.A., Otim B. (1982). Bovine Immune Response to Theileria Parva: Neutralizing Antibodies to Sporozoites. Immunology.

[6] Musoke A.J., Nantulya V.M., Rurangirwa F.R., Buscher G. (1984). Evidence for a Common Protective Antigenic Determinant on Sporozoites of Several Theileria Parva Strains. Immunology.

[7] Lacasta A., Mwalimu S., Kibwana E., Saya R., Awino E., Njoroge T., Poole J., Ndiwa N., Pelle R., Nene V. (2018). Immune Parameters to P67C Antigen Adjuvanted with ISA206VG Correlate with Protection against East Coast Fever. Vaccine.

[8] Musoke A., Morzaria S., Nkonge C., Jones E., Nene V. (1992). A Recombinant Sporozoite Surface Antigen of Theileria Parva Induces Protection in Cattle. Proc. Natl. Acad. Sci. USA.

[9] Nene V., Gobright E., Bishop R., Musoke A. (1999). Linear Peptide Specificity of Bovine Antibody Responses to p67 of *Theileria parva* and Sequence Diversity of Sporozoite-Neutralizing Epitopes: Implications for a Vaccine. Infect. Immun..

[10] Draper S.J., Angov E., Horii T., Miller L.H., Srinivasan P., Theisen M., Biswas S. (2015). Recent Advances in Recombinant Protein-Based Malaria Vaccines. Vaccine.

[11] Miura K. (2016). Progress and Prospects for Blood-Stage Malaria Vaccines. Expert. Rev. Vaccines.

[12] Miura K., Diouf A., Fay M.P., Barrett J.R., Payne R.O., Olotu A.I., Minassian A.M., Silk S.E., Draper S.J., Long C.A. (2023). Assessment of Precision in Growth Inhibition Assay (GIA) Using Human Anti-PfRH5 Antibodies. Malar. J..

[13] Liu K.-T., Han Y.-J., Wu G.-H., Huang K.-Y.A., Huang P.-N., Liu K.-T., Han Y.-J., Wu G.-H., Huang K.-Y.A., Huang P.-N. (2022). Overview of Neutralization Assays and International Standard for Detecting SARS-CoV-2 Neutralizing Antibody. Viruses.

[14] Tindih H.S., Geysen D., Goddeeris B.M., Awino E., Dobbelaere D.A.E., Naessens J. (2012). A Theileria Parva Isolate of Low Virulence Infects a Subpopulation of Lymphocytes. Infect. Immun..

[15] Baldwin C.L., Teale A.J., Naessens J.G., Goddeeris B.M., MacHugh N.D., Morrison W.I. (1986). Characterization of a Subset of Bovine T Lymphocytes That Express BoT4 by Monoclonal Antibodies and Function: Similarity to Lymphocytes Defined by Human T4 and Murine L3T4. J. Immunol..

[16] MacHugh N.D., Bensaid A., Howard C.J., Davis W.C., Morrison W.J. (1991). Analysis of the Reactivity of Anti-Bovine CD8 Monoclonal Antibodies with Cloned T Cell Lines and Mouse L-Cells Transfected with Bovine CD8. Vet. Immunol. Immunopathol..

[17] MacHugh N.D., Mburu J.K., Hamilton M.J., Davis W.C. (1998). Characterisation of a Monoclonal Antibody Recognising the CD3epsilon Chain of the Bovine T Cell Receptor Complex. Vet. Immunol. Immunopathol..

[18] Rocchi M.S.L., Ballingall K.T., MacHugh N.D., McKeever D.J. (2006). The Kinetics of Theileria Parva Infection and Lymphocyte Transformation in Vitro. Int. J. Parasitol..

[19] Rocchi M.S., Ballingall K.T., Ngugi D., MacHugh N.D., McKeever D.J. (2008). A Rapid and Sensitive Intracellular Flow Cytometric Assay to Identify Theileria Parva Infection within Target Cells. Parasitology.

[20] Toye P., Nyanjui J., Goddeeris B., Musoke A.J. (1996). Identification of Neutralization and Diagnostic Epitopes on PIM, the Polymorphic Immunodominant Molecule of Theileria Parva. Infect. Immun..

[21] Patel E., Mwaura S., Kiara H., Morzaria S., Peters A., Toye P. (2016). Production and Dose Determination of the Infection and Treatment Method (ITM) Muguga Cocktail Vaccine Used to Control East Coast Fever in Cattle. Ticks Tick Borne Dis..

[22] Lacasta A., Kim H.C., Kepl E., Gachogo R., Chege N., Ojuok R., Muriuki C., Mwalimu S., Touboul G., Stiber A. (2023). Design and Immunological Evaluation of Two-Component Protein Nanoparticle Vaccines for East Coast Fever. Front. Immunol..

[23] Makau M.C., Powell J., Prendergast J., Latré de Laté P., Morrison L.J., Fisch A., Gathura P., Kitala P., Connelley T., Toye P. (2020). Inverted CD4+/CD8+ T Cell Ratio in Boran (Bos Indicus) Cattle. Vet. Immunol. Immunopathol..

[24] Lu Y., Wang J., Li Q., Hu H., Lu J., Chen Z. (2021). Advances in Neutralization Assays for SARS-CoV-2. Scand. J. Immunol..

[25] Wallach M. (2010). Role of Antibody in Immunity and Control of Chicken Coccidiosis. Trends Parasitol..

[26] Lim S.S.Y., Chua K.H., Nölke G., Spiegel H., Goh W.L., Chow S.C., Kee B.P., Fischer R., Schillberg S., Othman R.Y. (2018). Plant-Derived Chimeric Antibodies Inhibit the Invasion of Human Fibroblasts by Toxoplasma Gondii. PeerJ.

[27] Fu Y.F., Feng M., Ohnishi K., Kimura T., Itoh J., Cheng X.J., Tachibana H. (2011). Generation of a Neutralizing Human Monoclonal Antibody Fab Fragment to Surface Antigen 1 of Toxoplasma Gondii Tachyzoites. Infect. Immun..

[28] Mineo J.R., McLeod R., Mack D., Smith J., Khan I.A., Ely K.H., Kasper L.H. (1993). Antibodies to Toxoplasma Gondii Major Surface Protein (SAG-1, P30) Inhibit Infection of Host Cells and Are Produced in Murine Intestine after Peroral Infection. J. Immunol..

[29] Nyagwange J., Tijhaar E., Ternette N., Mobegi F., Tretina K., Silva J.C., Pelle R., Nene V. (2018). Characterization of the Theileria Parva Sporozoite Proteome. Int. J. Parasitol..

