# Supplementary material for: An Improved Theileria parva Sporozoite Seroneutralization Assay for the Identification of East Coast Fever Immune Correlates
Source: Antibodies (Basel). 2024 Dec 5;13(4):100. doi: 10.3390/antib13040100 (PMC11672397; doi:10.3390/antib13040100)
Supplement: Supplementary file 1 [file antibodies-13-00100-s001.zip › antibodies-3217120-supplementary.pdf]

antibodies MDPI

SUPPLEMENTARY MATERIAL

Table S1: Average percentage of *T. parva* infected cells (and standard deviation) in the three sporozoite batches: 08/17, 09/17 and 10/17.

Average percentage of *T. parva* infected cells

	**Batch 8/17**			**Batch 9/17**			**Batch 10/17**		
**Days pi***	1/20	1/50	1/100	1/20	1/50	1/100	1/20	1/50	1/100
8	7.5 (3.2)	1.2 (0.5)	1.7 (1.4)	6.7 (1.4)	1.1(0.4)	1.9 (0.9)	8.6 (5.3)	2.6 (1.0)	1.8 (1.1)
9	11.0 (5.1)	1.6 (1.2)	2.0 (1.6)	4.8 (1.7)	4.0(1.5)	5.0 (3.0)	11.4 (4.3)	5.3 (3.3)	3.7 (0.8)
10	12.9 (6.7)	2.4 (1.6)	2.3 (1.3)	11.9 (3.7)	4.2(1.5)	1.4 (0.9)	12.9 (5.0)	7.1 (3.3)	4.2 (1.9)
11	14.3 (2.5)	5.6 (3.9)	2.4 (1.4)	17.7(3.1)	8.1 (3.7)	2.7 (1.7)	26.9 (5.7)	13.6 (1.6)	5.6 (4.0)
12	21.7 (5.0)	8.8 (5.0)	5.8 (4.0)	19.4(4.5)	8.9 (1.3)	5.9 (1.9)	24.0 (2.9)	14.3 (1.4)	5.9 (3.0)

pi: post-infection

Table S2. Neutralising capacity of antibodies was assessed individually and in combinations at two different dilutions, 10 and 1 µg/ml. The statistical significance of the differences between 10 and 1 µg/ml was calculated using a Mann-Whitney non-parametric test (significant differences are in bold).

**Antibody and antibody combinations**	**Median % neutralization (95% CI*)**		
	**10 µg/ml**	**1 µg/ml**	**p-value****
AR22.7 (anti-p67N)	24.49 (17.44-33.06)	13.50 (5.10-17.21)	**0.0106**
1A7 (anti-p67C)	26.20 (20.62-39.80)	16.20 (12.20-18.26)	**0.0356**
Bov-p67C	29.54 (22.86-35.36)	16.70 (9.79- 20.88)	**0.0055**
Combination AR22.7 + 1A7	18.73 (14.28-28.32)	13.34 (9.07-17.29)	0.1370
Combination Bov-p67C + 1A7	69.05 (54.30-78.17)	8.66 (2.11-19.57)	**<0.0001**
Combination Bov-p67C + AR22.7	28.78 (22.76-33.47)	11.34 (2.26-19.64)	**0.0110**

*CI: confidence interval

Table S3. Statistical analysis of differences in the neutralising capacity of individual and combinations of antibodies at 10 µg/ml, calculated using a Mann-Whitney non-parametric test.

**Antibody combinations**	1A7 (anti-p67C)	Bov-p67C	AR22.7+1A7	Bov-p67C+1A7	Bov-p67C+AR22.7
AR22.7 (anti-p67N)	0.6744	0.3810	0.4813	**0.0001**	0.6354
1A7 (anti-p67C)		0.8874	0.2276	**0.0004**	0.9636
Bov-p67C			0.1108	**0.0001**	0.8916
AR22.7 + 1A7				**<0.0001**	0.1471
Bov-p67C + 1A7					**0.0005**
Bov-p67C + AR22.7					
